# Lack of Matrilin-2 Favors Liver Tumor Development via Erk1/2 and GSK-3β Pathways *In Vivo*

**DOI:** 10.1371/journal.pone.0093469

**Published:** 2014-04-01

**Authors:** Alexandra Fullár, Kornélia Baghy, Ferenc Deák, Bálint Péterfia, Yvonne Zsák, Péter Tátrai, Zsuzsa Schaff, József Dudás, Ibolya Kiss, Ilona Kovalszky

**Affiliations:** 1 1st Department of Pathology and Experimental Cancer Research, Semmelweis University, Budapest, Hungary; 2 Institute of Biochemistry, Biological Research Center, Hungarian Academy of Sciences, Szeged, Hungary; 3 2nd Department of Pathology, Semmelweis University, Budapest, Hungary; 4 Department of Otorhinolaryngology, Medical University Innsbruck, Innsbruck, Austria; University of Patras, Greece

## Abstract

Matrilin-2 (Matn2) is a multidomain adaptor protein which plays a role in the assembly of extracellular matrix (ECM). It is produced by oval cells during stem cell-driven liver regeneration. In our study, the impact of Matn2 on hepatocarcinogenesis was investigated in *Matn2^-/-^* mice comparing them with wild-type (WT) mice in a diethylnitrosamine (DEN) model. The liver tissue was analyzed macroscopically, histologically and immunohistochemically, at protein level by Proteome Profiler Arrays and Western blot analysis. *Matn2^-/-^* mice exhibited higher susceptibility to hepatocarcinogenesis compared to wild-type mice. In the liver of *Matn2^-/-^* mice, spontaneous microscopic tumor foci were detected without DEN treatment. After 15 μg/g body weight DEN treatment, the liver of *Matn2^-/-^* mice contained macroscopic tumors of both larger number and size than the WT liver. In contrast with the WT liver, spontaneous phosphorylation of EGFR, Erk1/2 GSK-3α/β and retinoblastoma protein (p-Rb), decrease in p21/CIP1 level, and increase in β-Catenin protein expression were detected in *Matn2^-/-^* livers. Focal Ki-67 positivity of these samples provided additional support to our presumption that the lack of Matn2 drives the liver into a pro-proliferatory state, making it prone to tumor development. This enhanced proliferative capacity was further increased in the tumor nodules of DEN-treated *Matn2^-/-^* livers. Our study suggests that Matn2 functions as a tumor suppressor in hepatocarcinogenesis, and in this process activation of EGFR together with that of Erk1/2, as well as inactivation of GSK-3β, play strategic roles.

## Introduction

Matrilins are non-collagenous glycoproteins implicated in the organization of extracellular matrix (ECM) [1]. They can form homo-oligomers and assemble into filamentous networks, which are either connected to or independent of collagen fibrils. As matrilins can interact via their von Willebrand factor type A (vWFA) domains with various ECM components including proteoglycans and collagens, they are proposed to fulfill a bridging function in the ECM assembly of various tissues [2]. The matrilin family consists of four members (matrilin-1, -2, -3, -4). Matrilin-2 (Matn2) is the largest member with a minimum Mr of 104300 of the secreted monomer [3]. It is encoded by a gene spanning over 100 kb and transcribed from two promoters [4]. The Matn2 monomer is composed of two von Willebrand factor A-like domains, 10 epidermal growth factor-like modules, one unique sequence, and one coiled-coil domain [3]. Via the coiled-coil domain, Matn2 assembles into oligomers and can be detected as a mixture of monomers, dimers, trimers, and tetramers in tissue extracts and in the medium of cultured cells [5]. Matn2 can bind to fibrillar collagens, fibronectin and laminin-nidogen-1 complex [6]. Mann et al. found that integrin α1β1 does not play a major role in cellular interactions with matrilins. In the case of Matn2 a weak binding signal with soluble integrin α1β1 was seen, while the integrin α2β1 ectodomains did not show any binding [7]. However, the interaction between matrilins and integrins is comparatively weak, matrilins promote only weak cell attachment, and fail to trigger the formation of focal adhesions. It is not clear whether this weak interaction with integrins can activate signal transduction and induce gene expression [7], [8].

The matrilin-2 gene (*Matn2*) is expressed in a great variety of tissues, but at highly variable levels [3], [5]. The gene is transcribed and the protein is secreted by several established osteoblast, fibroblast, myoblast and epithelial cell lines. Matn2 mRNA and protein is also produced by hepatic oval cells, but not by hepatocytes [9]. In an experimental model of rat liver regeneration, Matn2 was deposited in the basement membrane zone around the tubules formed by oval cells, suggesting an important role for the protein in liver regeneration [9].

Furthermore, *Matn2* is expressed in certain tumors. Sporadic pilocytic astrocytoma, a pediatric brain tumor, is characterized by elevated MATN2 mRNA and protein level [10]. We also recently reported increased *MATN2* expression both at mRNA and protein levels in human liver cirrhosis and liver cancer [11]. While in normal human liver MATN2 is located in the walls of portal vessels, in hepatocellular carcinoma (HCC) it is intensively detected in the tumor vessels. However, it remains unknown how *MATN2* contributes to tumor formation.

Targeted disruption of *Matn2* in transgenic mice did not cause obvious phenotypic alterations [12]. *Matn2^-/-^* mice are viable, fertile and histological analyses did not reveal any obvious alterations in the architecture of mutant tissues at various embryonic and postnatal stages. However, no information is available about the consequences of Matn2 deficiency on tumor formation. To address this question here we investigated the role of Matn2 *in vivo* using an experimental mouse model of hepatocarcinogenesis. In Matn2-deficient livers we could demonstrate spontaneous appearance of tumorous foci, as well as markedly increased tumor formation upon diethylnitrosamine (DEN) treatment. Furthermore, we uncovered several alterations in signaling pathways likely to be involved in the pathomechanism of tumor formation.

## Materials and Methods

### 1. Animals and treatment

Inactivation of *Matn2* in embryonic stem cells and generation of inbred (129/SV) mouse strains have been described elsewhere [12]. *Matn2^+/−^* heterozygotes were mated to generate *Matn2^+/+^* (wild-type, WT) and *Matn2^-/-^* homozygotes for tumorigenesis. Fifteen-day old male WT and *Matn2^-/-^* mice were injected intraperitoneally with DEN at a dose of 15 μg/g body weight. Each group, including WT and *Matn2^-/-^* untreated controls as well as DEN-treated mice, consisted of ≥10 animals. Mice were terminated 10 months after DEN exposure by cervical dislocation in ether anesthesia. At termination, body weight and liver weight of the animals were measured and the number of macroscopically detectable tumors was counted.

All animal experiments were conducted according to the ethical standards of the Animal Health Care and Control Institute Csongrád County, Hungary. The protocol was approved by the Committee of the Animal Health Care and Control Institute Csongrád County, Hungary (permit No. XVI/03047-2/2008).

### 2. RNA extraction, reverse transcription and RT-PCR

RNA was isolated from frozen livers. After homogenization in liquid nitrogen the total RNA was isolated using the RNeasy Mini Kit (Qiagen, Hilden, Germany), according to the protocol provided by the manufacturer. The yield and purity of the isolated RNA was estimated by the ND-1000 spectrophotometer (NanoDrop Technologies, Wilmington, Delaware, USA). The integrity and size distribution of the total RNA was analyzed using Experion Automated Electrophoresis Station (Bio-Rad, Hercules, CA, USA).

cDNAs were generated from 1 μg of total RNA by M-MLV Reverse Transcriptase kit (Invitrogen by Life Technologies, Carlsbad, California, USA) following the instructions of the supplier. Real-time PCR (RT-PCR) was performed by ABI Prism 7000 Sequence Detection System (Applied Biosystems by Life Technologies, Welterstadt, Germany), using ABI Taqman Gene Expression Assays for β-actin (assay ID: Mm00607939_s1), Matn2 (assay ID: Mm01166023_m1) according to the manufacturer's protocol, mouse β-actin was used as endogenous control. All samples were run in duplicates in a 20 μl reaction volume with 50 ng of cDNA. Results were obtained as threshold cycle (CT) values. Expression levels were calculated by using the 2^−ΔC^_T_ method.

### 3. Phospho-MAPK and Phospho-RTK antibody arrays

For phosphoprotein antibody arrays and Western blot analysis proteins were isolated from frozen liver tissues. After homogenization in liquid nitrogen 1 ml of lysis buffer (20 mM TRIS pH 7.5, 2 mM EDTA, 150 mM NaCl, 1% Triton-X100, 0.5% Protease Inhibitor Cocktail (Sigma, St. Louis, MO,USA)) was added to the samples. After incubation for 30 min on ice, samples were centrifuged at 13000 rpm for 20 min. Supernatants were saved and protein concentrations were measured according to Bradford et al [13].

The activities of phospho-mitogen-activated protein kinase (phospho-MAPK) and phospho-receptor tyrosine kinase (phospho-RTK) were assessed by their relative levels of phosphorylation using the Proteome Profiler Array (R&D Systems, Minneapolis, MN, USA) according to the manufacturer's instructions. The same liver protein samples were used for Western blot. Pooled samples of three livers from the same experimental group were homogenized in lysis buffer (described above) and adjusted to 300 μg protein/250 μl lysate. Signals were developed by incubating the membrane in SuperSignal West Pico Chemiluminescent Substrate Kit (Thermo Fisher Scientific Inc., Waltham, MA USA), and visualized on a Kodak Image Station 4000MM Digital Imaging System.

### 4. Western blot analysis

Thirty μg of total proteins were mixed with loading buffer containing β-mercaptoethanol and were incubated at 95°C for 5 min. Pooled samples of three from the same experimental group were loaded onto a 10% polyacrylamide gel and were run for 30 min at 200 V on a Mini Protean vertical electrophoresis equipment (Bio-Rad, Hercules, CA, USA). Proteins were transferred to polyvinylidene fluoride (PVDF) membrane (Millipore, Billerica, MA, USA) by blotting overnight at 100 mA. Ponceau staining was applied to determine the efficiency of blotting. Membranes were blocked with 3% (w/v) non-fat dry milk (Bio-Rad) in TBS for 1 hour followed by incubation with the primary antibodies (p53, β-Catenin, glycogen synthase kinase 3 beta (GSK-3β), p-GSK-3α/β, p-c-Myc, extracellular signal-regulated kinases 1/2 (Erk1/2), p-Erk1/2, cyclin-dependent kinase inhibitor 1 (p21/CIP1), phospho-retinoblastoma (p-Rb), diluted 1∶500-1∶1000) at 4°C for 16 hours. Beta-actin served as loading control. Membranes were washed 5 times with TBS containing 0.5% (v/v) Tween20, then were incubated with appropriate secondary antibodies conjugated with HRP enzyme for 1 hour. Afterwards membranes were washed as before and signals were visualized with SuperSignal West Pico Chemiluminescent Substrate Kit (Thermo Fisher Scientific Inc.). Band densities were determined by Kodak Image Station 4000MM Digital Imaging System. Antibody data are provided in Supporting Information (Table S1).

### 5. Histological and immunohistochemical analysis

Liver samples were either frozen for further processing or fixed in 10% formaldehyde and embedded in paraffin for histological analysis. Paraffin sections were dewaxed in xylene and stained with hematoxylin and eosin (HE). Stained sections were used for histological diagnosis.

Immunofluorescence staining was performed on methanol-acetone-fixed 10-μm cryosections. Nonspecific binding was blocked first with 10% (w/v) normal donkey or goat serum in phosphate buffered saline (PBS) for 1 h. The specimens were incubated at 4°C overnight with primary antibodies (Matn2, laminin, β-Catenin, GSK-3β, p-GSK-3α/β, p-c-Myc diluted 1∶200-1∶400). After washing in PBS, sections stained for β-Catenin, GSK-3β, p-GSK-3α/β and p-c-Myc were incubated with Alexa Fluor 555 donkey anti-rabbit IgG secondary antibody (Invitrogen by Life Technologies) for 30 min at room temperature in a dark chamber. Nuclei were stained with 1 μg/ml 4′,6-diamidino-2-phenylindole (DAPI) in PBS for 5 min. Sections stained for Matn2 and laminin were washed and incubated with a combination of Cy3-conjugated donkey anti-goat IgG and Cy2-conjugated donkey anti-rabbit IgG antibodies, respectively (Jackson ImmunoResearch Laboratories Inc., West Grove, PA, USA). Pictures were taken by Nikon Eclipse E600 microscope with the help of Lucia Cytogenetics version 1.5.6 program or Bio-Rad MRC 1024 confocal laser microscope.

The antigen retrieval on the deparaffinized tissue sections was performed by Target Retrieval Solution (Dako, Glostrup, Denmark) in a pressure cooker under maximal pressure for 3 minutes, followed by proteinase K (0.5 mg/ml) digestion for 10 minutes. To inhibit endogenous peroxidases, samples were treated with 10% H_2_O_2_ in methanol for 30 minutes at room temperature. Normal serum was applied to block any nonspecific binding, and the slides were incubated with primary antibodies raised against specific proteins (p53, p-Erk1/2, p21/CIP1, Ki-67, p-Rb diluted 1∶75-1∶500) at 4°C overnight. Subsequently, the slides were incubated for 30 minutes with biotinylated goat anti-rabbit IgG secondary antibody at room temperature. A detailed list of the antibodies used in this study is presented in Supporting Information (Table S1). Signal amplification was made by avidin-biotin complex (ABC) (Vector Laboratories, Burlingame, CA USA) in a dilution of 1∶50 for 30 min at room temperature. The signal was detected with 3,3-diaminobenzidine tetrahydrochloride (DAB) substrate chromogen solution (Dako) followed by counterstaining with hematoxylin. Pictures were taken by MRC-1024 confocal laser scanning microscope (Hemel Hempstead, UK), Nikon Eclipse E600 fluorescent microscope (Tokyo, Japan), and Olympus BX50 (Tokyo, Japan) microscope.

### 6. Determination of Ki-67 index

After immunostaining of Ki-67 in liver sections of 5-5 animals, the number of immunopositive cells was counted in 10 fields of each slide at a magnification of 200× by Olympus BX50 (Tokyo, Japan) microscope.

### 7. Calculation of tumor volume

To determine the tumor volume of livers, HE stained sections were scanned by Panoramic Scan (3D Histech Ltd., Budapest, Hungary). The length and width of tumors in each section were determined with the help of Panoramic viewer program (3D Histech Ltd.) Tumor volume was calculated as V  =  (a^2^*b*π)/6 where ‘a’ refers to width (mm) and ‘b’ stands for length (mm).

### 8. Statistical analysis

All statistical analyses were made with Graphpad Prism 4.03 software (Graphpad Software Inc.). Data were tested for normal distribution by D'Agostino & Pearson's omnibus normality test. Significance of changes in WT control vs. WT DEN, WT control vs. *Matn2^-/-^* control, WT DEN vs. *Matn2^-/-^* DEN and *Matn2^-/-^* control vs. *Matn2^-/-^* DEN were tested by non-parametric tests (Mann-Whitney) and Students' t-tests depending on the distribution of the data. The independent experimental sets were then compared for reproducibility. Only reproducible significant changes were considered as „significant”. Significance was declared at the standard p<0.05 level.

## Results

### 1. Lack of Matn2 increases the proliferative capacity of liver cells

**Figure 1:**
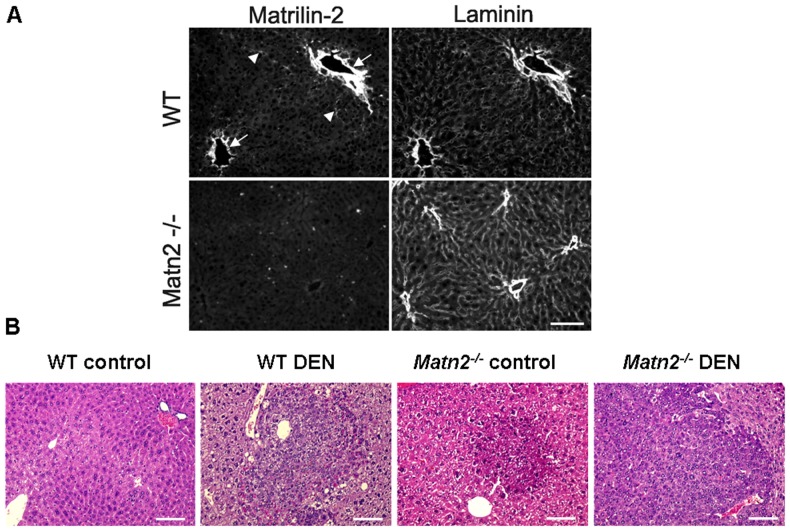
Immunolocalization of Matn2 in the liver of young mice (A). Matn2 immunostaining is most intense around the portal blood vessels (arrowhead) on a frozen section of a 40-day old WT mouse liver. Matn2 partially colocalizes with laminin; however, in several structures Matn2 staining is more intense (arrowheads). There is no immunosignal in the matching area in the liver of a *Matn2^-/-^* mice. Bar, 0.1 mm. **Representative histological stain of WT control, WT DEN-treated,** ***Matn2^-/-^*** **control and** ***Matn2^-/-^*** **DEN-treated mouse livers (B).** Scale bars represent 0.1 mm.

In the livers of young WT mice, Matn2 protein was clearly detected around the portal blood vessels, showing partial colocalization with laminin. No reaction was detectable in the livers of *Matn2^-/-^* mice, confirming the knockout phenotype of these animals (Figure 1A).

**Figure 2:**
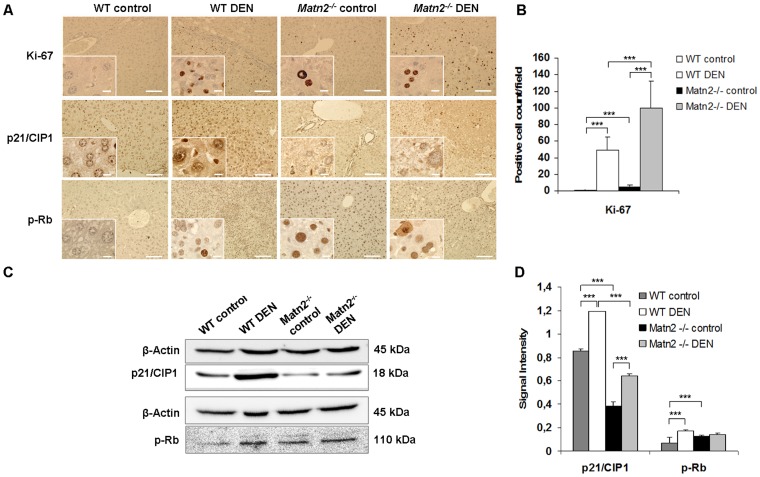
Representative immunohistochemical stains of WT control, WT DEN-treated, *Matn2^-/-^* control and *Matn2^-/-^* DEN-treated mouse livers (A). Scale bars represent 0.1(insets) for paraffin-embedded samples. Changes in cell cycle regulation. Ki-67 proliferation index (B). In knockout samples an average of 4.1 Ki-67 positive cells were counted per field of view compared to 0.7 in WT (p<0.001) (B). Results are expressed as mean ± SD. **Representative Western blots of cell cycle regulatory proteins (C) in WT control, WT DEN-treated,** ***Matn2^-/-^*** **control and** ***Matn2^-/-^*** **DEN-treated mouse livers.** Diagrams of band intensities expressed as values normalized to β-Actin loading control (D). Data are expressed as mean ± SD, n = 3.

Without any experimental challenge, neoplastic foci appeared in livers of *Matn2^-/-^* mice at the age of 10 months as seen in HE sections (Figure 1B). To confirm their proliferative capacity Ki-67 immunostaining was applied (Figure 2A), which showed a scattered pattern of positive cells in *Matn2^-/-^* liver sections but not in the WT livers. In knockout samples an average of 4.1 Ki-67 positive cells were counted per field compared to 0.7 in WT (p<0.001) (Figure 2B).

Next, p21/CIP1, a potent CDK inhibitor, and retinoblastoma (Rb) protein, two key regulatory molecule of the G1 cell cycle checkpoint were examined in whole liver homogenates. Western blot analysis revealed a ∼50% decrease in p21/CIP1 protein levels in *Matn2^-/-^* livers compared to WT samples (p<0.001) (Figure 2C, 2D). In parallel, phosphorylation of Rb at T780 was found to be significantly increased by 70% in *Matn2^-/-^* samples (p<0.001) (Figure 2C, 2D). In a good agreement with Western blot results, immunostaining of p21/CIP1 showed lower number of hepatocytes with positive nuclear reaction in *Matn2^-/-^* animals than in the WT (Figure 2A). Moreover, nuclei of *Matn2^-/-^* control livers reacted with phospho-Rb antibody, whereas the reaction was absent from WT livers (Figure 2A).

**Figure 3:**
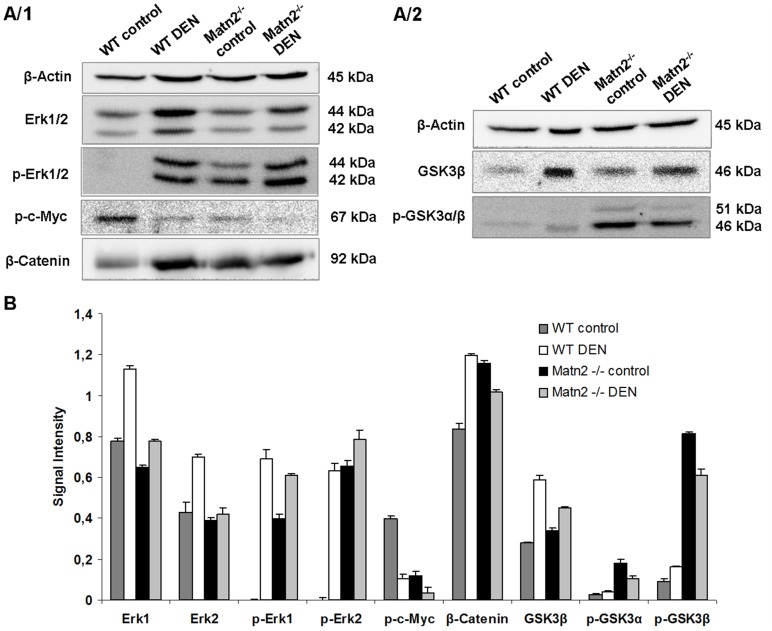
Representative Western blots of intracellular regulatory proteins in WT control, WT DEN-treated, *Matn2^-/-^* control and *Matn2^-/-^* DEN-treated mouse livers (**A/1, A/2**). Results of densitometrical analysis of band intensities expressed as values normalized to β-Actin loading control (**B**). Data are expressed as mean ± SD, n = 3.

**Figure 4:**
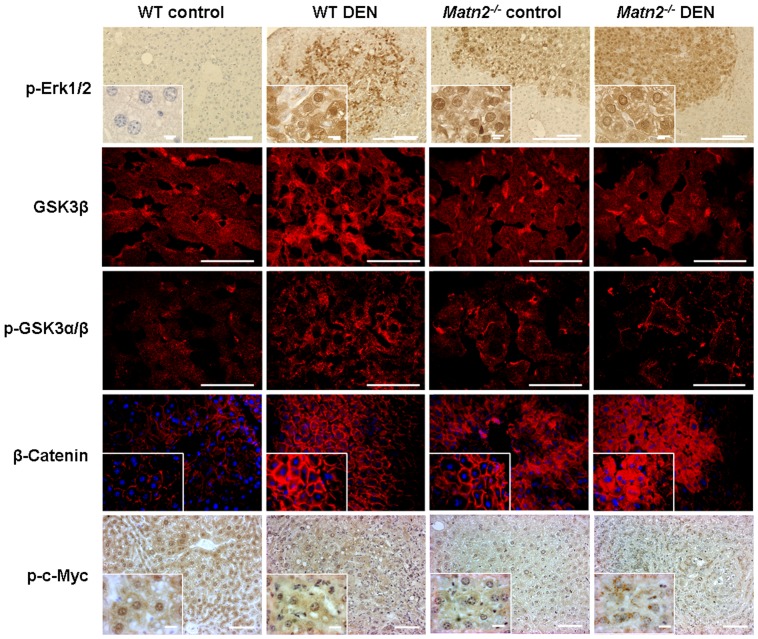
Representative immunofluorescence and immunohistochemical stains of WT control, WT DEN-treated, *Matn2^-/-^* control and *Matn2^-/-^* DEN-treated mouse livers. Scale bars represent 0.1(insets) for paraffin-embedded samples and 0.05 mm for frozen tissue sections.

To specify the signal transduction pathways involved in the activation of cell cycle, a phospho-MAPK array was applied (Figure S1) and validated by Western blots. Erk1/2, a member of the Ras/MAPK pathway, was spontaneously phosphorylated in *Matn2^-/-^* control livers as revealed by Western blot (Figure 3A/1, 3B). Immunostaining localized these activated cells into the hepatic foci (Figure 4).

Western blot indicated the outstanding role of GSK-3 proteins, too, in the activation of *Matn2^-/-^* hepatocytes. Both GSK-3 α and β exhibited higher phosphorylation levels at the classical inhibitory sites Ser9 and 21, respectively, in knockout livers compared to the WT (Figure 3A/2, 3B). In the case of p-GSK-3β a highly significant 9-fold elevation was observed, while the total amount of the protein did not show any notable change. Immunostaining of phospho-GSKs and total GSK-3β confirmed the results obtained by Western blot (Figure 4, 3A/2, 3B).

The amount of β-catenin, one of the key signaling molecules in the Wnt pathway, increased to 138% in untreated *Matn2^-/-^* mice compared to WT (Figure 3A/1, 3B). In parallel, fluorescent immunostaining revealed its translocation from the membrane to the cytoplasm of hepatocytes (Figure 4).

As GSK-3α/β is able to phosphorylate and thus inactivate c-Myc, this important proto-oncogene was also included in our investigation [14]. Indeed, phosphorylation of c-Myc at Thr58 residue was markedly lower in untreated *Matn2^-/-^* livers than in the corresponding WT ones (30% vs. 100%) (Figure 3A/1, 3B), probably reflecting impaired GSK-3β activity.

In summary, in untreated *Matn2^-/-^* livers GSK-3α/β becomes inactivated by phosphorylation and hence fails to phosphorylate c-Myc and β-catenin, thereby rescuing them from degradation. As a consequence, β-catenin translocates and accumulates in the cytoplasm, and c-Myc is allowed to act as a transcription factor in the nucleus (Figure 4).

**Figure 5:**
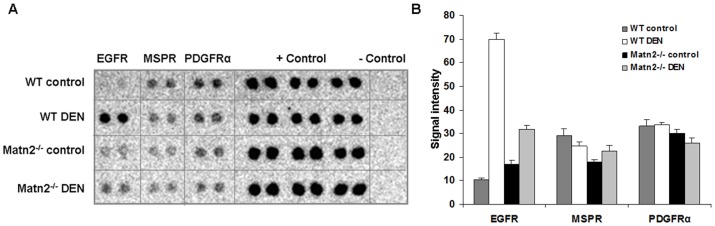
Results of Phospho-RTK antibody array. Picture of RTK array membrane (**A**). Densitometry of phosphorylation signals in WT control (dark gray bars) and WT DEN-treated (white bars) samples, compared to control (black bars) and *Matn2^-/-^* DEN-treated (light gray bars) samples (**B**). Data are expressed as mean ± SD, n = 3.

To identify potential transmembrane receptors that may transmit the altered signal from the extracellular matrix into the cells, a phospho-RTK array was carried out. The amount of active epidermal growth factor receptor (EGFR) was slightly higher in *Matn2^-/-^* control livers than in WT samples (Figure 5A, 5B).

Differences between the *Matn2^-/-^* and WT control groups with regard to signaling are summarized in Table 1.

**Table 1:** Differences between WT control and *Matn2^-/-^* control group compared to WT control.

	Total protein	Phosphorylation	Immunostaining	Localization
**EGFR**	-	↑	-	-
**Erk1/2**	↑↑↑	↑↑↑	↑↑	cytoplasm, nucleus
**GSK-3α/β**	↑	↑↑	↑	cytoplasm
**β-Catenin**	↑	-	↑	cytoplasm
**c-Myc**	-	↓ (inactivating)	-	cytoplasm, nucleus
**p21/CIP1**	↓	-	↓	nucleus
**Ki-67**	-	-	↑	nucleus
**Rb**	-	↑	↑↑	nucleus

### 2. Ablation of Matn2 results in enhanced tumor formation in experimental hepatocarcinogenesis

**Figure 6:**
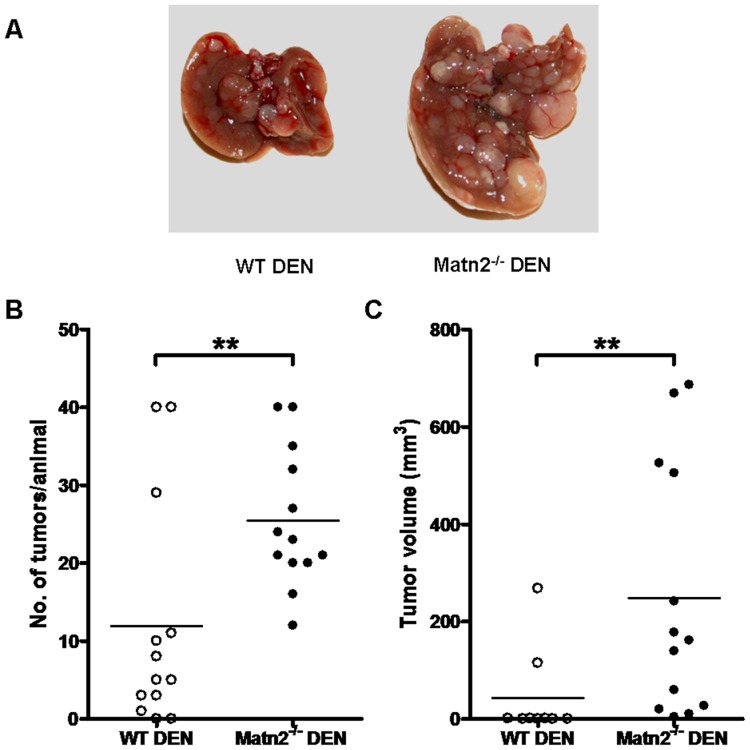
Results of DEN treatment in WT and *Matn2^-/-^* animals. Representative pictures of the macroscopic appearance of WT and *Matn2^-/-^* DEN-treated mouse livers. 10 months after DEN exposure the number and size of macroscopic tumors were greater in *Matn2^-/-^* than in WT livers (**A**). The number of tumors/animal (**B**) and the tumor volume (**C**) were significantly higher in *Matn2^-/-^* mice compared to WT mice after DEN-treatment (n = 9 for WT DEN, n = 13 for *Matn2^-/-^* DEN, **p<0.01).

To address the role of Matn2 in tumorgenesis, HCC in *Matn2^-/-^* and WT mice was induced by administering a single dose of DEN. DEN-exposed WT and *Matn2^-/-^* mice were compared to mock-injected littermates 10 months after treatment. DEN treatment induced macroscopic tumors in 78% of WT mice, whereas tumor frequency was 100% in the liver of *Matn2^-/-^* animals. A higher number of macroscopic tumors developed in *Matn2^-/-^* livers, with an average number of 23 tumors/liver in contrast with 4 counted in WT livers (p<0.01, Figure 6B). Moreover, not only the number but also the size of tumors was significantly larger in the livers lacking Matn2, (mean tumor volumes, 248 mm^3^ vs. 43 mm^3^, p<0.01) (Figure 6A, 6C).

#### a. Events of hepatocarcinogenesis in the presence of Matn2

In WT animals, DEN treatment initiated HCC formation with molecular changes already known from earlier studies of hepatocarcinogenesis. In DEN-provoked livers 15% higher matrilin-2 mRNA level was detected than that of untreated control livers (p<0.05) (Figure S2).

Whereas no immunopositivity for Ki-67 was detected in untreated WT liver sections, the reaction in DEN-exposed WT livers revealed a high number of immunopositive cells with an average count of 49/field (p<0.001 vs. WT control) (see Figure 2B). These cells were localized to the tumorous areas reflecting high proliferative activity. A marked ∼1.5-fold induction was detected in the levels of p21/CIP1(p<0.001), as well as a 2.3-fold increase in the amount of phospho-Rb (T780) (p<0.001) in WT DEN samples compared to WT control ones.

DEN treatment caused a ∼1.5-fold elevation of total Erk1/2 proteins compared to the untreated WT animals. Moreover, phosphorylation of these proteins was also significantly higher after DEN treatment than in controls, as seen both by immunostaining and Western blot (Figure 4, 3A/1, 3B).

The inactive phosphorylated forms of GSK-3α/β were increased in tumor-bearing livers. Compared to the normal livers a 60% elevation for α and 80% for β was measured by densitometry of Western blots (Figure 3B). In parallel, total GSK-3β showed a 2-fold increment in WT DEN samples (Figure 3B). Immunohistochemical studies confirmed these observations, too (Figure 4).

Compared to WT controls, inhibition of GSK activity resulted in 1.5× higher amount of β-catenin and 70% less c-Myc phosphorylation in WT DEN liver homogenates, as revealed by Western blots (Figure 3B). Immunostaining of β-catenin demonstrated its translocation from the cell membrane and accumulation in the cytoplasm. A loss of p-c-Myc was also evident by immunohistochemistry (Figure 4).

By phospho-RTK array, significantly higher phospho-EGFR levels were detected in the homogenates from DEN-treated WT wild-type animals compared to control ones (Figure 5A, 5B). No changes in macrophage stimulating protein receptor (MSPR) and platelet-derived growth factor receptor (PDGFR) phosphorylation were seen.

Differences between WT control and WT DEN groups are summarized in Table 2.

**Table 2:** Differences between WT control and WT DEN group compared to WT control.

	Total protein	Phosphorylation	Immunostaining	Localization
**EGFR**	-	↑↑↑	-	-
**Erk1/2**	↑↑	↑↑↑	↑↑↑	cytoplasm, nucleus
**GSK-3α/β**	↑↑	↑	↑	cytoplasm
**β-Catenin**	↑	-	↑	cytoplasm
**c-Myc**	-	↓↓	-	cytoplasm, nucleus
**p21/CIP1**	↑↑	-	↑↑	nucleus
**Ki-67**	-	-	↑↑↑	nucleus
**Rb**	-	↑	↑	nucleus

#### b. The lack of Matn2 causes changes similar to DEN treatment in the liver of WT animals

Table 1 shows molecular differences between untreated WT and *Matn2^-/-^* animals; Table 2 summarizes differences between untreated and DEN-treated WT mice. Comparison of these two tables reveals remarkable similarities. For EGFR, Erk1/2, GSKs, β-Catenin, p-c-Myc, Ki-67, and p-Rb, the direction and magnitude of changes are nearly identical between WT control vs. *Matn2^-/-^* control and WT control vs. WT DEN groups. Our data indicate that *Matn2^-/-^* hepatocytes harbor alterations that make them prone to malignant transformation even without hepatocarcinogenic challenge; indeed, *Matn2^-/-^* mice developed spontaneous foci in the liver. Only a single protein, p21/CIP1, behaved differently in these comparisons. p21/CIP1 was induced upon DEN treatment in WT livers; however, the lack of Matn2 seemed to interfere with the induction of p21/CIP1.

#### c. Alterations in the liver of Matn2-deficient mice after HCC induction

As mentioned previously, Matn2-deficient mice were more sensitive to DEN treatment, displaying higher number of tumors with significantly larger volume in their livers compared to the WT DEN group (see Figure 6A–C). We also described that *Matn2^-/-^* livers had an activated, pro-proliferatory phenotype without any experimental challenge.

When compared to *Matn2^-/-^* control, 24.4 times more Ki-67 immunopositive cells were counted in sections of matrilin-2 deficient livers after DEN treatment (Figure 2B). In parallel, a 1.6-fold increase in p21/CIP1 levels was detected in *Matn2^-/-^* DEN homogenates relative to *Matn2^-/-^* controls (p<0.01) (Figure 3C, 3D). Among the intracellular signaling molecules, a 1.5-fold elevation in p-Erk1/2 and a 65% decrease in p-c-Myc levels were observed by Western blot, and confirmed by immunostaining (Figure 4, 3A/1, 3B). Furthermore, slightly lower levels of β-Catenin and p-GSKα/β were detected (Figure 4, 3A/1, 3A/2, 3B), and phospho-EGFR displayed a 1.9-fold increase (Figure 5A, 5B) in *Matn2^-/-^* DEN samples as compared to *Matn2^-/-^* controls. These molecular changes are summarized in Table 3.

**Table 3:** Differences between *Matn2^-/-^* control and *Matn2^-/-^* DEN group compared to *Matn2^-/-^* control.

	Total protein	Phosphorylation	Immunostaining	Localization
**EGFR**	-	↑	-	-
**Erk1/2**	↑	↑	↑ =	cytoplasm, nucleus
**GSK-3α/β**	↑	↓	= ↓	cytoplasm
**β-Catenin**	↓	-	↑	cytoplasm
**c-Myc**	-	↓	-	cytoplasm, nucleus
**p21/CIP1**	↑	-	↑	nucleus
**Ki-67**	-	-	↑↑↑	nucleus
**Rb**	-	= ↑	=	nucleus

As seen in Table 3, DEN treatment did not lead to robust molecular alterations in *Matn2^-/-^* livers. The elevation of Ki-67 was the most prominent, as the tumors developed upon HCC induction had a high rate of cell division. In conclusion, since the lack of Matn2 had already driven hepatocytes into an activated state, DEN treatment caused only modest intensification of pro-proliferative signaling.

When comparing groups of WT and *Matn2^-/-^* mice both treated with DEN, higher proliferation rate was seen in *Matn2^-/-^* DEN livers, as the average Ki-67-positive cell count was 49 in WT and 100 in *Matn2^-/-^* livers after DEN exposure (Figure 2B). In parallel, 46% less p21/CIP1 protein was present in DEN-treated *Matn2^-/-^* livers as compared to DEN-treated WT (p<0.001) (Figure 2C, 2D). With regard to intracellular signaling molecules, p-GSK-3α/β exhibited 2.4- vs. 3.8-fold increase, respectively, in *Matn2^-/-^* vs. WT upon DEN treatment (Figure 3A/2, 3B) and, accordingly, in *Matn2^-/-^* DEN the levels of p-c-Myc were only 37% of those measured in WT DEN (Figure 3A/1, 3B). Conversely, the active phospho-form of Erk2 was markedly, by 20%, higher in *Matn2^-/-^* DEN relative to WT DEN (Figure 3A/1, 3B). However, the amount of active p-EGFR in *Matn2^-/-^* DEN was as low as 45% of that measured in WT DEN (Figure 5A, 5B).

Differences between WT DEN and *Matn2^-/-^* DEN groups are summarized in Table 4. This comparison suggests that the underlying mechanism behind the higher number and volume of tumors experienced in DEN-treated *Matn2^-/-^* livers may involve enhanced GSK-3β inactivation and hindered p21/CIP1 induction, leading to elevated proliferation rate indicated by Ki-67.

**Table 4:** Differences between WT DEN and *Matn2^-/-^* DEN group compared to WT DEN.

	Total protein	Phosphorylation	Immunostaining	Localization
**EGFR**	-	↓	-	-
**Erk1/2**	↓	↑	=	cytoplasm, nucleus
**GSK-3α/β**	↓	↑↑	↓	cytoplasm
**β-Catenin**	↓	-	↑	cytoplasm
**c-Myc**	-	↓	-	cytoplasm, nucleus
**p21/CIP1**	↓↓	-	↓	nucleus
**Ki-67**	-	-	↑↑	nucleus
**Rb**	-	↓ =	↑	nucleus

## Discussion

Despite the relatively detailed data available on matrilin-2 structure and tissue expression, its function has not yet been elucidated [3], [5], [15], [16]. Matn2 is an ECM protein, which was found to be expressed in the liver progenitor oval cells [9]. Only one report implicated MATN2 in human HCC, but without analysis of Matn2-related signaling [11]. In the current study a functional analysis was applied using the well-established DEN-induced HCC model [12]. The induction of macroscopic tumors required 10 months in both WT and *Matn2^-/-^* mice. In matrilin-2-deficient animals, however, significantly more, and more voluminous tumor foci have developed. Furthermore, quite surprisingly, sporadic microscopic tumor foci spontaneously appeared in the liver of *Matn2^-/-^* mice without DEN treatment at age of 10 months. This result evidenced that the lack of Matn2 triggers an activated state in the liver, making it prone to tumor development. Our observations suggest a protective role for *Matn2* against liver carcinogenesis. To explore the underlying mechanisms, activation of key signal transduction proteins in *Matn2^-/-^* animals has been investigated in more detail.

The most conspicuous finding in *Matn2^-/-^* livers was increased phosphorylation of Erk1/2. Activation of the MAPK pathway most likely was a critical event related to the absence of Matn2 from the ECM, and this might be one of the key alterations responsible for the increased susceptibility of hepatocytes to cancer development. In addition, inactivation of GSK-3α/β proteins was revealed by Western blot. This implies that beside Erk1/2 activation the signal generated in the absence of Matn2 utilizes the Wnt pathway and inactivates GSK-3β and -3α by phosphorylation at Ser9 and Ser21 residues. Although it is mostly protein kinase B (Akt protein) that inactivates GSK-3β [17], and phospho-MAPK array in our study indicated slightly increased phosphorylation of p38, pan Akt, and Akt3, we could not confirm this result on Western blot. On the other hand Ding and coworkers published that Erk1/2 is also capable of priming GSK-3β for inactivation by phosphorylating it at the T43 residue [18]. Conversely, it has also been reported that inhibition of GSK-3β significantly induces phosphorylation of Erk1/2 through PKCδ activation [19].

In accordance with the inactivation of GSK-3β we were able to demonstrate an increase in β-catenin protein levels, and a decrease in the inactivating phosphorylation of c-Myc at the Thr58 residue. Suspension of GSK-3β-mediated inhibitory effects brings hepatocytes into a proliferative state [20]. Indeed, accelerated cell cycling in untreated *Matn2^-/-^* mice was confirmed by immunostaining for Ki-67. Whereas β-catenin in the WT control livers was confined to the cell membrane, the protein accumulated in the cytoplasm in *Matn2^-/-^*, but no nuclear localization was ascertained. Currently we cannot explain why β-catenin failed to translocate to the nucleus, since its localization is thought to be solely determined by the availability and phosphorylation of its binding partners. This aspect of the mechanism needs further investigation [21].

Beside the β-catenin-related mechanism, Jin et al. reported that GSK-3β has a critical role in the phosphorylation-mediated inactivation of the Cyclin-dependent kinase 4/CyclinD3 complex (Cdk4/CyclinD3). This offers another approach how the inactivation of GSK-3β may result in increased proliferation [22].

Our results regarding p21/CIP1 fit very well into the model outlined. p21/CIP1 levels increased upon DEN-treatment in WT mouse liver, which is in line with the available reports [23]. In contrast, lack of Matn2 resulted in a decrease of p21/CIP1 levels in the liver, and DEN induction failed to reverse this tendency. This implies that a Matn2-facilitated signaling may be essential for the upregulation of p21/CIP1 in DEN-induced liver carcinogenesis. Our results demonstrating the inactivation of GSK-3β and a consequent decrease in the phosphorylation of c-Myc can explain this finding, as c-Myc is known to induce AP4 which serves as an inhibitory transcription factor of p21/CIP1 [24].

Erk1/2 activation in the context of DEN treatment has been reported to increase the expression and stability of p21/CIP1 [25]. This stabilized p21/CIP1 might be at least partly functional, resulting in relatively attenuated tumor formation in the DEN-treated WT mouse liver. Indeed, we could observe p21/CIP1 upregulation in the liver of DEN-treated WT mice at the protein level. Such upregulation upon DEN treatment was also seen in the *Matn2^-/-^* animals; however, this induction failed to compensate for the initially lower p21/CIP1 levels in *Matn2^-/-^*. Hence, the absolute amount of p21/CIP1 remained lower in the DEN-treated *Matn2^-/-^* mice, too. In *Matn2^-/-^* animals, activation of GSK-3β/c-Myc/p21/CIP1 axis may result in transcriptional inhibition of p21/CIP1 synthesis. The above-mentioned Erk1/2-mediated stabilization of p21/CIP1 is also likely to be Matn2-dependent [25]. Other mechanisms probably involved in p21/CIP1 protein inactivation include calreticulin-induced inhibition of translation [26], miR-106-induced inactivation, or degradation by the proteasomal system [27].

Increased phosphorylation of Rb protein at Ser780 in *Matn2^-/-^* indicates that, as a consequence to p21/CIP1 downregulation, Rb becomes inactivated by a Cdk4/CyclinD3-mediated process [22]. In addition, Erk1/2 has been reported to associate with the Cdk4/CyclinD1 complex and facilitate its function [28]. This facilitation may also lead to increased p-Rb phosphorylation in the liver of *Matn2^-/-^* mice, since the ablation of Matn2 was shown to be associated with elevated Erk1/2 activity.

To find the link between altered ECM composition and modified signaling, tyrosine kinase receptor phosphorylation was assessed. This revealed increased phosphorylation of EGFR only. Considering that Matn2 contains several EGF-like domains it can be hypothesized that Matn2 as a decoy molecule may interfere with the binding of the real EGF ligand to its receptor. This could imply that the lack of Matn2 increases the binding capacity of EGFR to its real ligand, resulting in increased activation. However, this hypothesis requires further studies to prove. Also, despite our efforts, integrin receptors possibly involved in mediating the effects of Matn2 could not so far be identified.

## Conclusion

**Figure 7:**
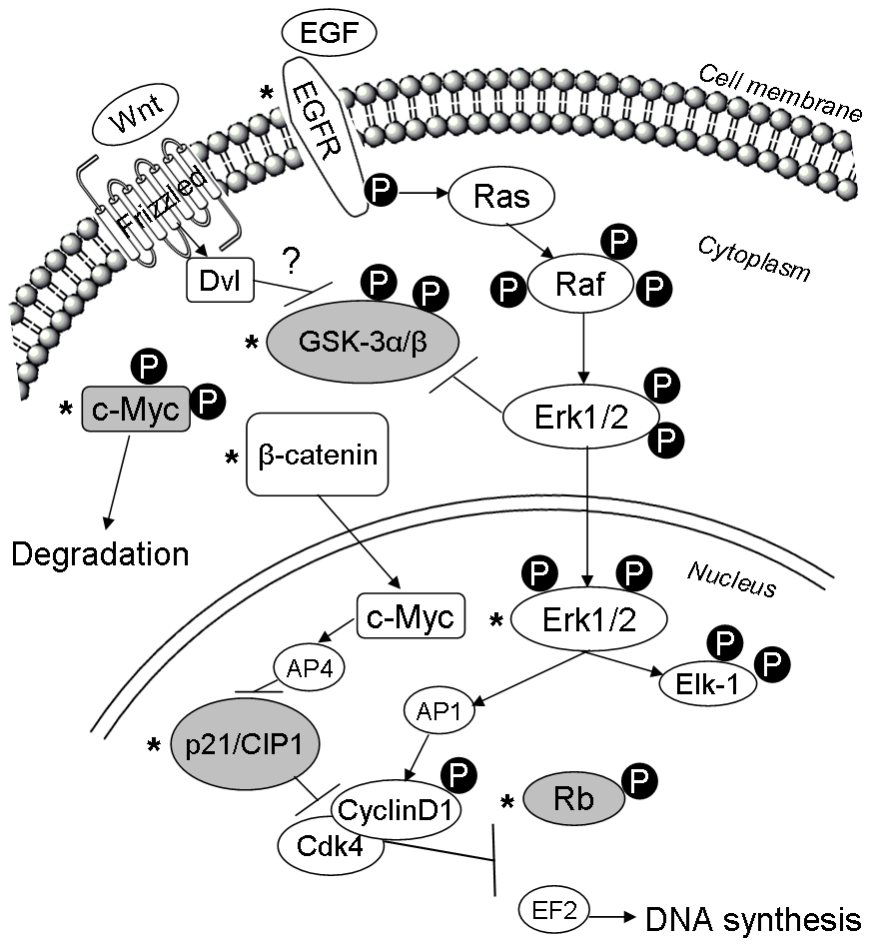
Schematic illustration of the predicted signaling mechanisms in *Matn2^-/-^* mice. Molecules studied in this work are labeled by asterisks. Empty boxes show upregulation and/or activation; gray boxes indicate downregulation and/or inactivation of the protein.

This study provides evidence that Matn2 functions as a tumor suppressor in hepatocarcinogenesis. *Matn2^-/-^* animals showed increased susceptibility to cancer induction, and spontaneously developed atypical microscopic foci in the liver. Ablation of Matn2 is thought to contribute to hepatocarcinogenesis through 1) activation of the MAPK signaling pathway; 2) inactivation of GSK-3β with consequent increase in the amount of β-catenin and activation of c-Myc; and 3) lowered levels of p21/CIP1 and consequent Rb phosphorylation by Cdk4/CyclinD complexes (Figure **7**).

## Supporting Information

Figure S1 **Phospho-MAPK antibody array to assess the activity of downstream signal transduction pathways.** Densitometry of phosphorylation signals in WT control (dark grey bars) and WT DEN-treated (white bars), compared to control (black bars) and *Matn2^-/-^* DEN-treated samples (light grey bars).
(TIF)

Figure S2 **Matn2 mRNA expression in wild type control and DEN-exposed livers detected by real-time RT-PCR.** As seen, 1.15-times more Matn2 mRNA was detected in tumorous samples (WT DEN) compared to control ones (WT control). Data are expressed as mean ± SD, n = 10; *p<0.05.
(TIF)

Table S1 **Antibodies used in the present study.**
(DOC)
