# Supplementary figures and images for: Lack of Matrilin-2 Favors Liver Tumor Development via Erk1/2 and GSK-3β Pathways In Vivo
Source: PLoS One. 2014 Apr 1;9(4):e93469. doi: 10.1371/journal.pone.0093469 (PMC3972106; doi:10.1371/journal.pone.0093469)

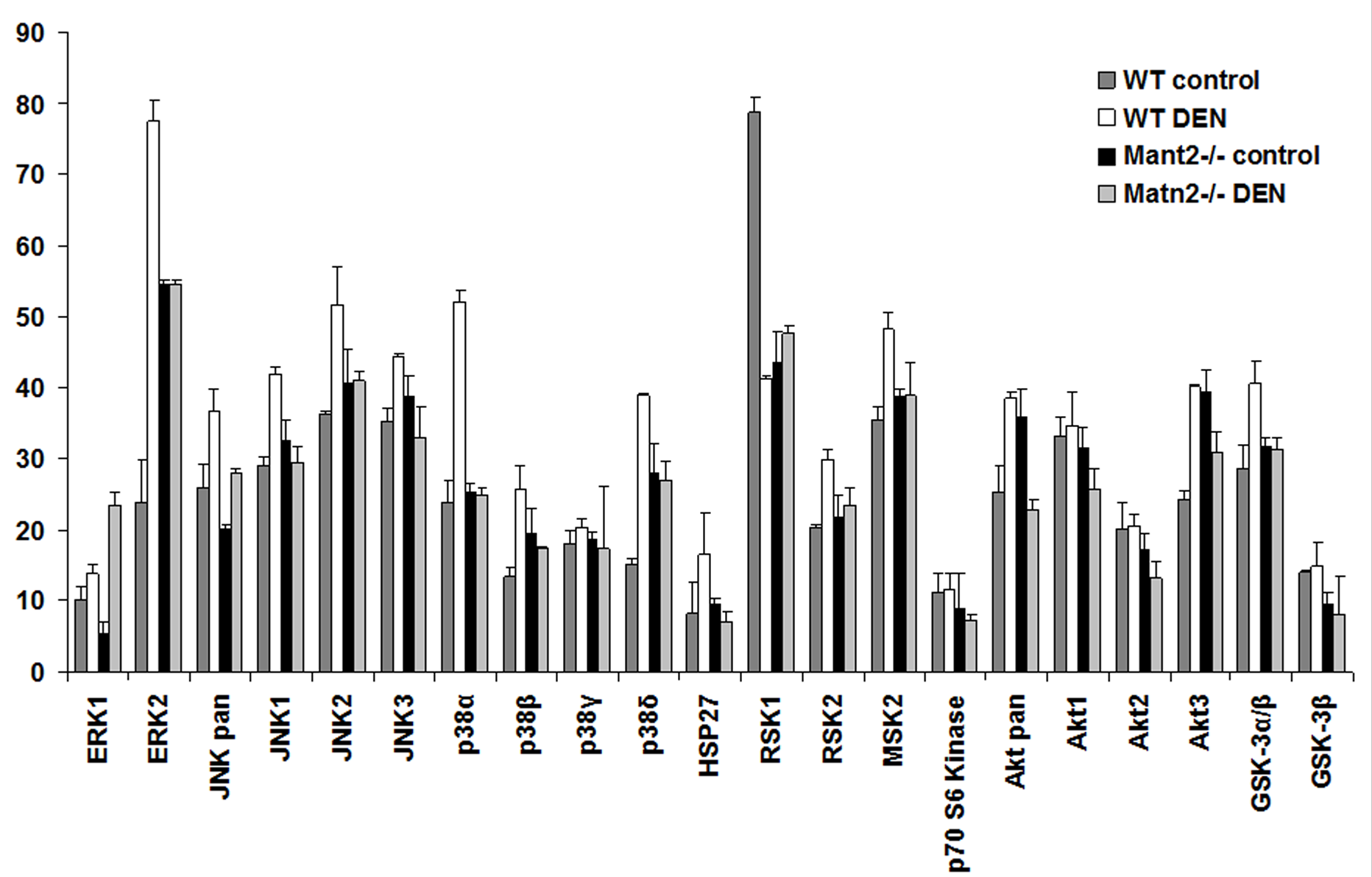

Supplement: Figure S1 — Phospho-MAPK antibody array to assess the activity of downstream signal transduction pathways. Densitometry of phosphorylation signals in WT control (dark grey bars) and WT DEN-treated (white bars), compared to control (black bars) and Matn2-/- DEN-treated samples (light grey bars). (TIF) [file pone.0093469.s001.tif]

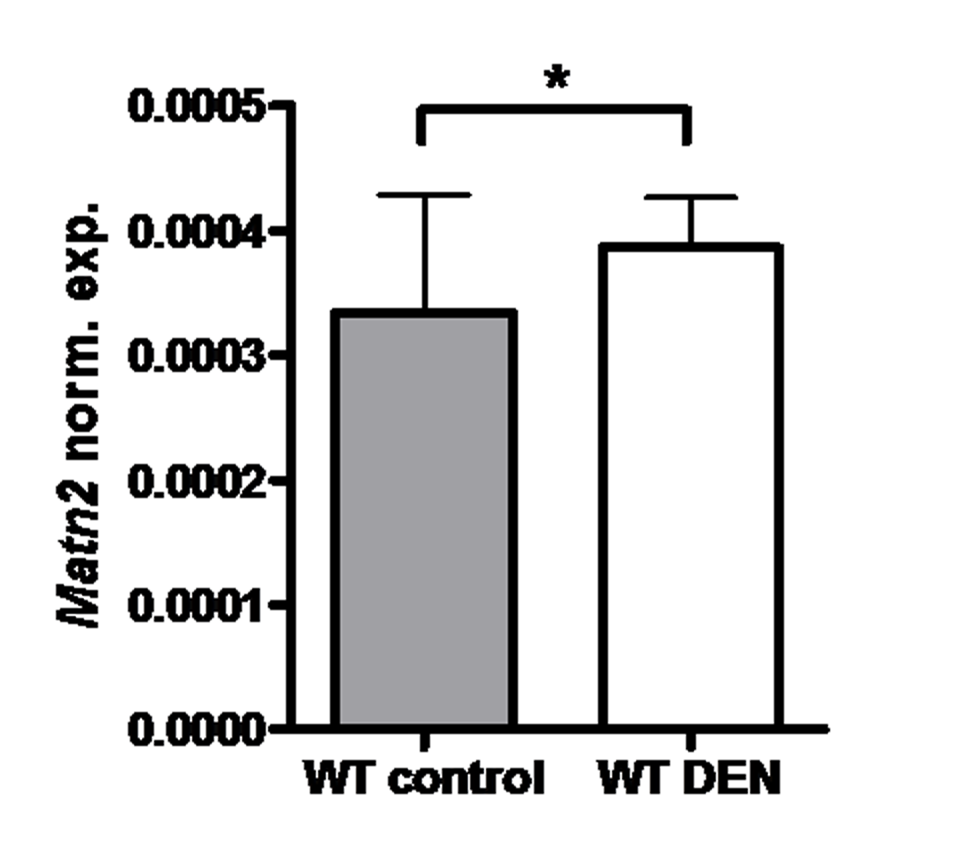

Supplement: Figure S2 — Matn2 mRNA expression in wild type control and DEN-exposed livers detected by real-time RT-PCR. As seen, 1.15-times more Matn2 mRNA was detected in tumorous samples (WT DEN) compared to control ones (WT control). Data are expressed as mean ± SD, n = 10; *p<0.05. (TIF) [file pone.0093469.s002.tif]
