# Supplementary material for: Lack of Matrilin-2 Favors Liver Tumor Development via Erk1/2 and GSK-3β Pathways In Vivo
Source: PLoS One. 2014 Apr 1;9(4):e93469. doi: 10.1371/journal.pone.0093469 (PMC3972106; doi:10.1371/journal.pone.0093469)
Supplement: Table S1 — Antibodies used in the present study. (DOC) [file pone.0093469.s003.doc]

| **Primary antibodies** | **Species** | **Manufacturer*** | **Cat. No.** | **Dilution for IHC and IF** | **Dilution for WB** |
| --- | --- | --- | --- | --- | --- |
| β-Actin Antibody | Rabbit monoclonal | Cell Signaling Technology, Danvers, MA | 4967 | - | 1:1000 |
| β-Catenin Antibody | Rabbit polyclonal | Cell Signaling Technology, Danvers, MA | 9562 | 1:200 | 1:1000 |
| p53 Antibody | Rabbit polyclonal | Abcam, Cambridge, UK | ab61256 | 1:75 | 1:500 |
| p21 Antibody | Rabbit polyclonal | Abcam, Cambridge, UK | ab7960 | 1:75 | 1:500 |
| Phospho-Rb (Ser780) Antibody | Rabbit polyclonal | Cell Signaling Technology, Danvers, MA | 9307 | 1:500 | 1:750 |
| p44/42 MAP Kinase | Rabbit polyclonal | Cell Signaling Technology, Danvers, MA | 9102 | - | 1:500 |
| Phospho-p44/42 MAP  Kinase (Thr202/204) | Rabbit monoclonal | Cell Signaling Technology, Danvers, MA | 4370 | 1:100 | 1:1000 |
| Ki-67 Antibody | Rabbit monoclonal | Thermo Fischer Scientific Inc. Fremont, CA | RM-9106 | 1:2 | - |
| GSK-3β (27C10) | Rabbit monoclonal | Cell Signaling Technology Danvers, MA | 9315 | - | 1:500 |
| Phospho-GSK-3α/β (Ser 21/9) Antibody | Rabbit polyclonal | Cell Signaling Technology Danvers, MA | 9331 | 1:100 | 1:750 |
| Anti-Phospho-c-Myc (Thr58) | Rabbit polyclonal | Thermo Scientific, Pierce Biotechnology, Rockford, USA | PA1-14268 | 1:200 | 1:500 |
| Matrilin-2 | Goat polyclonal | R&D Systems, Minneapolis, MN | AF3234 | 1:200 | - |
| Laminin from yolk sac tumor | Rabbit polyclonal | DakoCytomation, Glostrup, Denmark | Z0009 | 1:400 | - |
| **Secondary antibodies** | **Species** | **Manufacturer*** | **Cat. No.** | **Dilution for IHC** | **Dilution for WB** |
| Anti-goat  immunoglobulins/  Biotinylated | Rabbit polyclonal | DakoCytomation, Glostrup, Denmark | E 0466 | 1:200 | - |
| Anti goat IgG  Cy2-conjugated | Donkey polyclonal | Jackson ImmunoResearch Laboratoies Inc., West Grove, PA | 711-225-152 | 1:400 | - |
| Anti rabbit IgG  Cy3-conjugated | Donkey polyclonal | Jackson ImmunoResearch Laboratoies Inc., West Grove, PA | 705-165-147 | 1:400 | - |
| Alexa Fluor® 555 anti-rabbit IgG | Donkey polyclonal | Invitrogene by Life Technologies, Carlsbad, California | A31572 | 1:200 | - |
| Anti-goat  immunoglobulins/HRP | Rabbit polyclonal | DakoCytomation  Glostrup Denmark | P 0449 | - | 1:2000 |
